# Curriculum Consistency Learning and Multi-Scale Contrastive Constraint in Semi-Supervised Medical Image Segmentation

**DOI:** 10.3390/bioengineering11010010

**Published:** 2023-12-22

**Authors:** Weizhen Ding, Zhen Li

**Affiliations:** Department of Computer and Information Engineering, School of Science and Engineering, The Chinese University of Hong Kong (Shenzhen), Shenzhen 518000, China; 115010133@link.cuhk.edu.cn

**Keywords:** medical image segmentation, semi-supervised learning, curriculum learning, consistency loss, contrastive learning

## Abstract

Data scarcity poses a significant challenge in medical image segmentation, thereby highlighting the importance of leveraging sparse annotation data. In addressing this issue, semi-supervised learning has emerged as an effective approach for training neural networks using limited labeled data. In this study, we introduced a curriculum consistency constraint within the context of semi-supervised medical image segmentation, thus drawing inspiration from the human learning process. By dynamically comparing patch features with full image features, we enhanced the network’s ability to learn. Unlike existing methods, our approach adapted the patch size to simulate the human curriculum process, thereby progressing from easy to hard tasks. This adjustment guided the model toward improved convergence optima and generalization. Furthermore, we employed multi-scale contrast learning to enhance the representation of features. Our method capitalizes on the features extracted from multiple layers to explore additional semantic information and point-wise representations. To evaluate the effectiveness of our proposed approach, we conducted experiments on the Kvasir-SEG polyp dataset and the ISIC 2018 skin lesion dataset. The experimental results demonstrated that our method surpassed state-of-the-art semi-supervised methods by achieving a 9.2% increase in the mean intersection over union (mIoU) for the Kvasir-SEG dataset. This improvement substantiated the efficacy of our proposed curriculum consistency constraint and multi-scale contrastive loss.

## 1. Introduction

Accurate pixel-level labeling of medical images is essential for various applications such as clinical evaluation, therapy, and surgical planning in the field of medical image segmentation. However, the process of annotating precise labels for these images is both time-consuming and expensive. Consequently, obtaining a substantial amount of high-quality labeled data proves to be a challenging task. In contrast, there is a wealth of un-labeled medical data that are readily available. Leveraging this vast pool of un-labeled medical images holds great significance in addressing the scarcity of annotated data. To overcome the challenges posed by limited labeled data, semi-supervised learning has emerged as a promising approach. This technique effectively utilizes a combination of a small amount of labeled data and a larger volume of un-labeled data for training neural networks. By leveraging the vast amounts of un-labeled medical data, semi-supervised learning provides a valuable means to alleviate the scarcity of annotated medical images.

Previous methods based on convolutional neural networks have achieved excellent performance in medical image segmentation [1,2,3]. However, the hunger for high quality and large amounts of annotated segmentation data limits these methods. Semi-supervised learning aims to mix up a small number of annotated datasets and a large number of un-labeled images to train a segmentation model [4,5,6]. Bai [7] applied a pseudo-label method for cardiac image segmentation. Zhao [8] designed a cross-level contrastive learning method, which improved the representation capacity of local features. References [9,10,11,12] achieved advancements in semi-supervised medical image analysis by utilizing uncertainty measures. References [13,14] improved the models’ visual representations using contrastive learning techniques. However, previous methods have focused on implementing a static training scheme for semi-supervised medical segmentation, thereby causing the models to focus on specific features and leaving them weak in robustness. Therefore, we explored the dynamic learning process for medical image segmentation.

Curriculum learning is an algorithm inspired by human learning behavior patterns that can be widely applied to various deep learning algorithms. Generally, the human education process is organized from junior concepts and is gradually evolved into senior concepts. Traditional algorithms train target models using disorder training data, thereby ignoring the feature complexity of training samples and the convergence state of the current model during training. Specifically, curriculum learning divides the original dataset into sub-datasets of different difficulties, and the model starts to learn from easier sub-datasets. As training progresses, harder sub-datasets are updated to the training set, and the difficulty of the training task gradually increases to the final target task. In addition, curriculum learning is a plug-in method and has demonstrated effective performance in computer vision and natural language processing.

**Figure 1:**
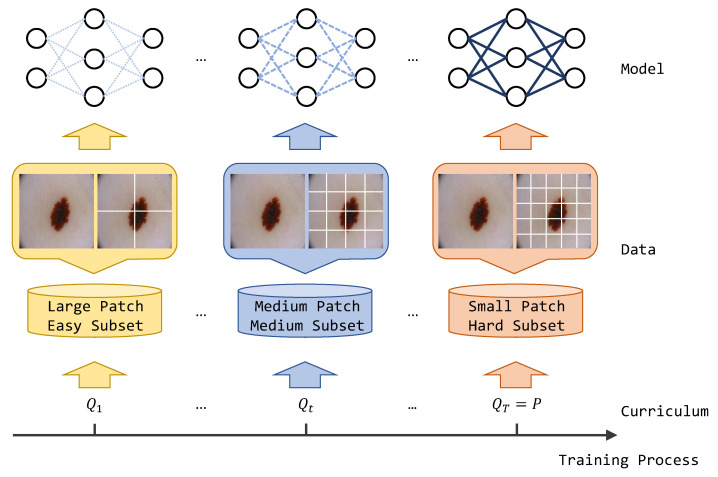
The motivation and the core idea of this paper. We propose a curriculum consistency learning scheme from the image and dynamically cropped patches, thus guiding the model coverage to a better optima in semi-supervised medical image segmentation.

There have been numerous studies in the past that have applied curriculum learning to medical image segmentation tasks. Hoel [15] designed a curriculum model based on additive regression, which assisted in improving segmentation results by predicting label attributes such as region size and the centroid position of the target as auxiliary tasks. Wang [16] introduced a multi-task curriculum strategy, where the auxiliary decoder learned image-level information to enhance the model’s pixel-level prediction. Liu [17] proposed the use of a style transfer model to generate curriculum samples of varying difficulty, thereby aiming to improve the model’s overall generalization ability. Nartey [18] applied curriculum learning in 3D CT image segmentation. In contrast to previous methods, we present a novel curriculum training strategy based on self-supervised learning for semi-supervised medical image segmentation. Unlike utilizing an auxiliary regression model, our approach incorporates a dynamic patch transformation technique that simulates the human curriculum learning process, thus progressing from easy to hard examples. This dynamic training strategy guides the model to learn diverse feature representations of the target, thus resulting in improved segmentation outcomes. Additionally, we introduce a multi-scale contrastive loss that enhances the representation capacity of cross-level semantic feature relations, thereby further refining the segmentation results. The motivation of our method is shown in Figure 1. For the convenience of the research community, we have made the source codes available at https://github.com/hkjcpy/Curriculum-Consistency-Learning accessed on 2 June 2023. Our contributions can be summarized as follows:

- Curriculum consistency constraint: We introduced a curriculum consistency constraint in the field of semi-supervised medical segmentation. By leveraging the inherent structure of the curriculum learning framework, our method optimized the model to converge at a better optima with increased generalization. This constraint facilitated the learning process and improved the model’s performance.
- Multi-scale contrastive loss: We applied a multi-scale contrastive loss that focused on promoting the representation capacity of cross-level semantic feature relations. This loss function enhanced the model’s ability to capture and leverage contextual information, thereby resulting in more accurate and refined segmentation results.
- Experimental evaluation: We evaluated our proposed method on two widely used datasets, namely, the polyp dataset Kvasir-SEG and the skin lesion dataset ISIC 2018. Through comprehensive experiments, we demonstrated that our approach surpassed other existing semi-supervised methods in terms of segmentation accuracy and performance. This highlights the efficiency and effectiveness of the proposed algorithm in tackling the challenges of semi-supervised medical image segmentation.

Overall, our work contributes to the advancement of semi-supervised medical image segmentation by introducing a novel curriculum training strategy and a multi-scale contrastive loss. The superior results achieved on benchmark datasets underscore the potential of our approach in improving the accuracy and reliability of medical image segmentation, thus benefiting clinical applications and supporting medical professionals in their diagnostic and treatment planning processes.

## 2. Materials and Methods

### 2.1. Materials

Polyp dataset Kvasir-SEG and skin lesion dataset ISIC 2018 were used for evaluating our approach. We compared our model’s performance with existing state-of-the-art semi-supervised methods such as UAMT [19], URPC [20], CCT [21], and CLCC [8].

Kvasir-SEG dataset [22]: A comprehensive dataset that includes a collection of gastro-intestinal polyp images, which are accompanied by their corresponding segmentation masks and bounding boxes. Gastro-intestinal polyps are abnormal tissue growths that develop within the mucous membrane lining the gastro-intestinal tract. Detecting and characterizing polyps is of paramount importance, as they can potentially be cancerous or precursors to cancerous growths. Manual image segmentation poses challenges, including the tedium, time consumption, and the potential for physician bias and inter-observer variation. To ensure the highest quality annotations, a team consisting of an engineer and a medical doctor meticulously outlined the margins of all polyps in the 1000 images. These annotations were further reviewed and validated by an experienced gastro-enterologist, thereby adding an extra layer of expertise to the process. Additionally, to leverage segmentation masks, bounding boxes were labeled to accurately represent the polyp regions. The enhanced dataset included polyp images, segmentation masks, and bounding boxes. The regions of interest (ROIs) in these images represented the pixels depicting polyp tissue, and they were depicted as a white foreground in the segmentation masks. The ROIs were generated through meticulous manual annotations, which were carefully verified by an experienced gastro-enterologist. Furthermore, the bounding boxes provided a set of coordinates that accurately enclosed the polyp regions.

ISIC 2018 dataset [23]: This is an extensive public repository of dermo-scopic images of skin data. Skin cancer is a prevalent and costly disease in the United States, with extremely expensive care expenditure. Timely detection plays a crucial role in improving patient outcomes, particularly for melanoma, which is the most lethal form of skin cancer. When detected early, the five-year survival rate for melanoma can reach an impressive 99% However, delayed diagnosis significantly reduces this rate. In an effort to combat this issue, the International Skin Imaging Collaboration (ISIC) has organized the largest skin image analysis challenge worldwide. Through this initiative, the ISIC has curated an extensive public repository of dermoscopic images of skin, which comprise 2594 images with corresponding ground truth segmentation masks. Dermoscopy involves examining skin lesions using specialized magnification and lighting techniques, which aide in the early detection and diagnosis of skin cancer.

### 2.2. Related Works

Several studies have explored the application of curriculum learning in the context of medical image segmentation. This section reviews some notable works in this area.

Liu et al. proposed a novel framework called “Style Curriculum Learning for Robust Medical Image Segmentation” [17]. The authors addressed the challenge of distribution shifts in image intensities between the training and test datasets. They introduced a unique style curriculum that trained segmentation models in an easy-to-hard mode, thus ensuring robust segmentation, even in the presence of distribution shifts.

Kervadec conducted a study titled “Curriculum Semi-supervised Segmentation” [15], where a curriculum-style strategy was investigated for semi-supervised CNN segmentation. The authors developed a regression network that learned image-level information, such as the size of the target region. This information effectively regularized the segmentation network, thereby constraining the softmax predictions of un-labeled images to match inferred label distributions.

In the paper “A Curriculum Domain Adaptation Approach to the Semantic Segmentation of Urban Scenes” [24], the authors proposed a curriculum-style domain adaptation method for the semantic segmentation of urban scenes. They employed easy and useful tasks, such as inferring label distributions for target images and landmark superpixels in the curriculum. This approach aimed to gain necessary properties regarding the target domain, thereby enhancing the segmentation performance.

Another relevant work is “Multi-Task Curriculum Learning for Semi-Supervised Medical Image Segmentation” [16]. The authors introduced a novel multi-task semi-supervised segmentation algorithm with a curriculum-style learning strategy. Their approach involved a segmentation task and an auxiliary regression task. The auxiliary task focused on learning image-level properties, including the size and centroid position of the target region. These properties served as regularization cues, thereby ensuring that the pixel-level segmentation results matched the distributions obtained from the regressions.

To improve the quality of pseudo-labeling in the context of semi-supervised semantic segmentation, the paper “Pruning-Guided Curriculum Learning for Semi-Supervised Semantic Segmentation” [25] proposed a novel method. The authors addressed the ambiguity of confidence scores by leveraging network pruning. They refined the confidence scores by considering the impact of pruning on prediction accuracy, thus enhancing the generalization ability of the network.

Additionally, the work “FlexMatch: Boosting Semi-Supervised Learning with Curriculum Pseudo Labeling” [26] introduced curriculum pseudo-labeling (CPL), which is a curriculum learning approach for leveraging un-labeled data. CPL dynamically adjusted thresholds for different classes based on the model’s learning status, thus allowing informative un-labeled data and their pseudo-labels to be utilized. This method did not introduce additional parameters or computations, thus making it computationally efficient.

In “An Efficient Semi-Supervised Framework with Multi-Task and Curriculum Learning for Medical Image Segmentation” [27], the authors proposed a semi-supervised segmentation framework using multi-task curriculum learning. The authors integrated a main segmentation task with two auxiliary tasks: feature regression and target detection. The auxiliary tasks predicted simpler image-level attributes and bounding boxes, which served as pseudo-labels for the main segmentation task. This approach ensured that the pixel-level segmentation results aligned with the distributions of these pseudo-labels.

These studies mentioned above have explored various aspects of curriculum learning for medical image segmentation, including robustness against distribution shifts, semi-supervised scenarios, domain adaptation, network pruning, and multi-task learning. These works provide valuable insights and techniques for advancing the field of medical image segmentation.

### 2.3. Methods

**Figure 2:**
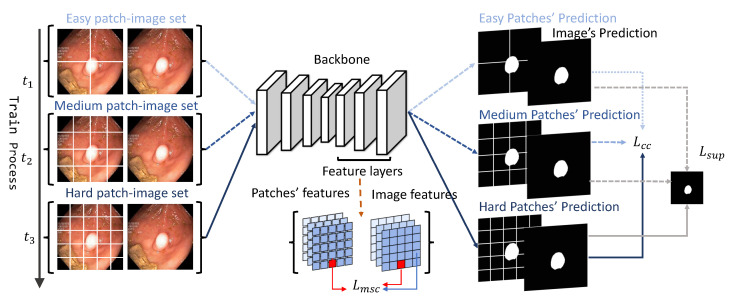
The overview pipeline of the proposed method.

Given an annotated image set $D^{l}=\left\{\left({x_{i}}^{l},y_{i}^{l}\right)\right\}$ and an un-labeled image set $D^{u}=\left\{{x_{i}}^{u}\right\}$, the goal of semi-supervised segmentation is to leverage both labeled and un-labeled data to boost the model. The overview of the proposed framework is illustrated in Figure 2. More details about the curriculum consistency learning scheme and multi-scale contrastive loss are introduced in the following sections.

#### 2.3.1. Curriculum Consistency Constraint

The core idea of the consistency-based approach is that the model should make similar predictions of un-labeled images with data augmentations. Unlike previous consistency methods, which implement static perturbations, we proposed a novel patch image consistency loss that dynamically controlled the difficulty of consistency learning for facilitating model convergence and boosting segmentation performance. Specifically, a complete image and its corresponding dynamically cropped patches were sent into the model for global–local prediction comparison. As the training epoch increased, the difficulty of the global–local comparison gradually increased, thereby guiding the model to learn the representational features from easy to harder levels. Notably, we used U-Net [28] as the segmentation backbone. Given a medical image $x\in R^{H\times W\times 3}$, a global feature map $Z_{g}$ will be extracted by a U-net extractor. Simultaneously, we decomposed the input image *X* into a set of non-overlapped patches $P=\{x_{i}\in R^{\frac{H}{\sqrt{m_{t}}}\times \frac{W}{\sqrt{m_{t}}}\times 3}\}$, where $m_{t}$ is the patch number that is dynamically adjusted by the curriculum scheme. Then, we input these patches *P* to the same backbone to extract local feature maps $\left\{z_{d}^{i}\right\}$. Finally, we sent both the global and local feature maps to segmentation layers $F_{seg}$ to calculate the consistency loss. In our experiments, we defined a curriculum trigger set—$\left\{n_{t},n=1,2,3,\ldots \right\}$ to dynamically control the decomposition size. For *t*-th epoch training, the curriculum consistency loss $L_{cc}$ is defined via Equation (1):

(1) $$\begin{array}{c} L_{cc}=\sum_{i=1}^{m(t)}MSE\left(F_{seg}\left(Z_{g}\right),F_{seg}(z_{i})\right)\\ m\left(t\right)=\sum_{j}I\left(t_{j}<t<t_{j+1}\right)\times n_{t}. \end{array}$$

Specifically, in our experiment, we designed three phases for curriculum learning. We set $n_{1}$, $n_{2}$, and $n_{3}$ to be 4, 16, and 25, respectively. Correspondingly, m(t) represents the number of cropped patches during training process, which was set to 4, 16, and 25 in the first, second, and third phases of training, respectively. When m(t) is small, each patch contains more visual information, thus resulting in lower difficulty in achieving consistency in segmentation predictions. The model can learn to infer basic properties under low-difficulty tasks. As m(t) increases, the difficulty of achieving consistency in segmentation predictions increases with less visual information remaining in each patch. Therefore, the model is forced to facilitate more accurate segmentation prediction under the high-difficulty tasks.

#### 2.3.2. Multi-Scale Contrastive Loss

The curriculum consistency loss can enhance the similarity of the image patch prediction. In addition, in order to improve the representation capacity of the semantic features among different levels, we applied an un-supervised multi-scale contrastive loss. Particularly, for a full image *X* and the corresponding patch set $\left\{x_{i}\right\}$, we extracted the corresponding global feature set $\left\{Z_{g}^{k},k=1,2,3,\ldots \right\}$ and patch feature sets $\left\{{\left\{z_{m}^{i}\right\}}^{k},k=1,2,3,\ldots \right\}$ from layers of different depths. By projecting them into a representation space, the representive features of patches are forced to be similar to the represent features of the whole image. The multi-scale contrastive loss $L_{msc}$ is formulated as follows:

(2) $$\begin{array}{c} L_{msc}=\sum_{k}L_{contrast}\left(k\right)\\ L_{contrast}\left(k\right)=-\sum_{i}log\frac{exp[{\left(F_{proj}\left(Z_{g}^{k}\right)\right)}_{i}\times F_{proj}\left(z_{m}^{i}\right)]}{exp\left[{\left(F_{proj}\left(Z_{g}^{k}\right)\right)}_{i}\times F_{proj}\left(z_{m}^{i}\right)\right]+\sum_{j\neq i}exp\left[{\left(F_{proj}\left(Z_{g}^{k}\right)\right)}_{i}\times {\left(F_{proj}\left(Z_{g}^{k}\right)\right)}_{j}\right]}, \end{array}$$

where $F_{proj}$ is the corresponding projection head for the *k*-th feature layer; ${\left(F_{proj}\left(Z_{g}^{k}\right)\right)}_{i}$ is the corresponding *i*-th position of $F_{proj}\left(Z_{g}^{k}\right)$; $F_{proj}\left(z_{m}^{i}\right)$ is treated as a positive pair of ${\left(F_{proj}\left(Z_{g}^{k}\right)\right)}_{j}$, where j≠i are treated as negative pairs for contrastive learning. Then, we summed the contrastive loss through all the positions and took the average. For more details, we used one projection head to project original features from one decoder layer of a certain depth. Since we applied multi-scale features of different layers, we used different projection heads to gain the projected features from different depths. Then, we calculated the respective contrastive losses and added them together to obtain the $L_{msc}$.

#### 2.3.3. Loss Function

The overall algorithm is illustrated in Algorithm 1. And the overall loss function *L* is illustrated in Equation (3), which contains three parts: un-supervised curriculum consistency loss $L_{cc}$ , multi-scale contrastive loss $L_{msc}$, and a combined supervised loss $L_{sup}$:

(3) $$\begin{array}{c} L=\alpha L_{cc}+\beta L_{msc}+L_{sup}\\ L_{sup}=\frac{1}{2}\left(CE\left(F_{p}\left(Z\right),Y\right)+Dice\left(F_{p}\left(Z\right),Y\right)\right), \end{array}$$

where *α* and *β* control the two-stage training process. In the first stage, *α* and *β* are set to be 0 and 1, respectively, and the multi-scale contrastive loss and supervised loss are activated to enhance the representation capacity. In the next stage, *α* and *β* are set to be 1 and 0, respectively. The curriculum consistency loss is utilized to strengthen the model to capture details and converge to a better optima. The total training epochs were 300, with 70 epochs for the first stage and the rest of theepochs for the second stage. In the second stage, we set curriculum set N = [4,16,25] and [$t_{1},t_{2},t_{3}$] to [80,160,300], respectively.
**Algorithm 1:** Medical image segmentation via curriculum consistency learning and multi-scale contrastive constraint.**Notation**: Curriculum Feature Extractor $E_{cur}$; Global Feature Extractor $E_{glob}$; Multi-scale Feature Extractor $E_{ms}$; Projection Layer $F_{proj}$; Segmentation Layer $F_{seg}$;**Input**: Labeled Images $D^{l}=\left\{\left({x_{i}}^{l},y_{i}^{l}\right)\right\}$; Unlabeled Images $D^{u}=\left\{{x_{i}}^{u}\right\}$**Output**: Segmentation Prediction $\hat{Y}$**1** //Training Phase**2** For training epoch t = 0,1,...,n **do****3**  //Supervised Training**4**  For $\left(x_{i},y_{i}\right)$ in $D^{l}$**5**   $Z_{g}⟵E_{glob}\left(x_{i}\right)$**6**   Minimize Loss $L_{sup}=\frac{1}{2}\left(CE\left(F_{seg}\left(Z_{g}\right),y_{i}\right)+Dice\left(F_{seg}\left(Z_{g}\right),y_{i}\right)\right)$**7**  //Unsupervised Training**8**  For $x_{i}$ in $D^{u}$**9**   $Z_{d}⟵E_{cur}\left(x_{i},t\right)$**10**   $Z_{m}⟵E_{ms}\left(x_{i},t\right)$**11**   $Z_{g}⟵E_{glob}\left(x_{i}\right)$**12**   Minimize Loss $\alpha L_{cc}\left(F_{seg}\left(Z_{g}\right),F_{seg}(Z_{d})\right)+\beta L_{msc}\left(F_{proj}\left(Z_{g}\right),F_{proj}(Z_{m})\right)$**13** End**14** //Inference Phase**15** $\hat{Y}⟵F_{seg}\left(E_{glob}(X)\right)$

## 3. Results

### 3.1. Experiments Details

Our implementation of the proposed method was based on the PyTorch [29] framework. All models were trained for a total of 300 epochs using the AdamW optimizer [30] with a learning rate of 10^−3^ and a default weight decay. The training process was conducted on an Nvidia A100 GPU. To ensure a balanced representation of the labeled and un-labeled data during training, we maintained a 1:1 sampling probability for both types of data. However, the actual data size for labeled and un-labeled data differs. In the Kvasir-SEG dataset, the ratio of labeled to un-labeled data is 1:4, while in the ISIC 2018 dataset, the ratio is 1:9. To form each training batch, we set a batch size of eight, which consisted of four labeled images and four un-labeled images. To ensure consistency across all the methods, we used the same dataset, backbone model, supervised loss function, and number of training epochs. Before training, all images were resized to a resolution of 320 × 320 pixels. This consistent pre-processing step helps to maintain uniformity and ensures fair comparisons between different methods. To evaluate the performance of each method, we randomly selected labeled images three times for training and recorded the mean and standard deviation of the metrics. The metrics used for evaluation included the mean absolute error (MAE), Dice coefficient, and mean intersection over union (mIoU). These metrics provided valuable insights into the accuracy and quality of the segmentation results produced by each method. By utilizing these evaluation metrics and conducting multiple trials, we could robustly assess the performance of our proposed method and compare it to other existing methods in the field of semi-supervised medical image segmentation.

### 3.2. Measure Metrics

We used the mean square error (*MSE*), mean intersection over union (*mIoU*), and the Dice coefficient in Equation (4) to evaluate our performance. We applied the proposed approach through comparison experiments with other SOTA semi-supervised methods. The experiment results demonstrated the better performance of our approach.

The mean square error (*MSE*) is a commonly used metric for quantifying the average squared difference between the predicted pixel-wise label and ground truth across an image.The *MSE* is expressed as the mean of these squared differences, thus providing a single numerical value that represents the average squared error. A lower value indicates a lower degree of difference.

The Dice coefficient is a widely accepted metric that is used to assess the correspondence between predicted segmentation and the ground truth on a pixel-wise level. It serves as a standard measure for comparing the results of segmentation algorithms. In this evaluation, Pred represents the predicted set of pixels, while GT represents the ground truth delineation of the object within the image.The Dice coefficient provides a similarity measure ranging between 0 and 1. It quantifies the level of agreement between the predicted and ground truth segmentations. A value closer to one indicates a higher degree of similarity, thereby suggesting a stronger alignment between the two sets.

The mean intersection over union (*mIoU*) is another standard metric that is used to evaluate a segmentation method. The *mIoU* calculates the Jaccard index similarity between Pred and its corresponding ground truth GT. The *mIoU* also provides a similarity measure ranging between 0 and 1. A higher value closer to one indicates a higher degree of similarity.

### 3.3. Metric Results Analysis

Table 1 presents the results of the semi-supervised segmentation on the Kvasir-SEG dataset, which demonstrate the superior performance of our method compared to the previous state-of-the-art (SOTA) approach. Our method exhibited a significant improvement, with a 1.93% decrease in the mean absolute error (MAE), a 9.2% increase in the mean intersection over union (mIoU), and a 7.8% increase in the Dice coefficient. These results indicate the effectiveness of our proposed approach in achieving superior segmentation performance.

To further evaluate the contributions of each component within our approach, we conducted an ablation study, whose results are shown in Table 1. The ablation study allowed us to assess the impact of each component by selectively removing them. Specifically, ‘Our w/o $L_{cc}$’ refers to the ablation study without the curriculum consistency loss, while ‘Our w/o $L_{msc}$’ refers to the ablation study without the multi-scale contrastive loss. The metric degradation observed in the ablation experiments served as empirical evidence of the effectiveness of each proposed component.

In addition, Table 2 presents the segmentation results for the ISIC 2018 dataset. Similar to the results for the Kvasir-SEG dataset, our method outperformed previous semi-supervised methods, thus further highlighting the superiority of the curriculum consistency learning and multi-scale contrastive constraint. To measure the effectiveness of each proposed component, we conducted a similar ablation study, thereby allowing us to assess their individual contributions.

(4) $$\begin{array}{cccc} MAE & =\frac{1}{n}\sum_{i=1}^{n}\left|Pred-GT\right|,\;mIoU & =\frac{1}{n}\sum_{i=1}^{n}\frac{\left|Pred\cap GT\right|}{\left|Pred\cup GT\right|},\;Dice & =\frac{2|Pred\cap GT|}{\left|Pred|+|GT\right|}. \end{array}$$

## 4. Discussion

**Figure 3:**
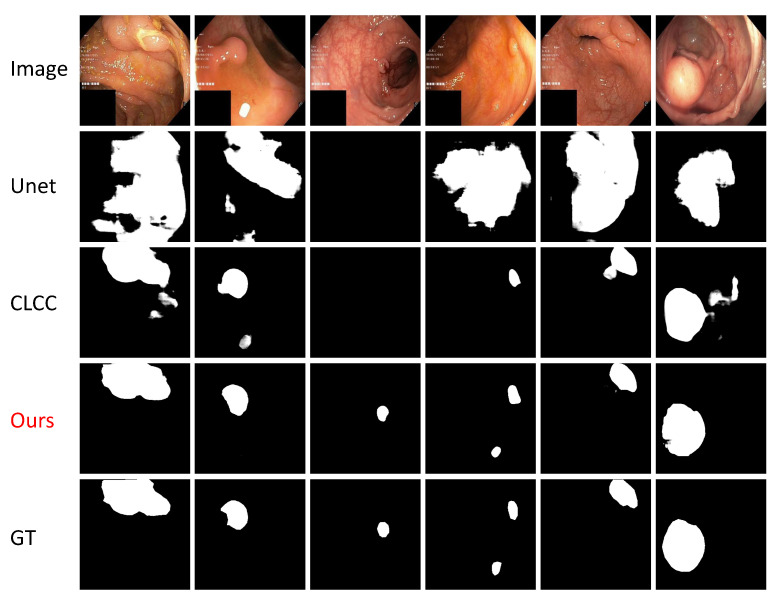
Qualitative comparison between our approach and CLCC method for the Kvasir-SEG test dataset.

Figure 3 showcases the qualitative outcomes of our method for the Kvasir-SEG test dataset, thereby highlighting the notable contrast between our approach and the previous state-of-the-art (SOTA) method, the CLCC. In comparison to the CLCC, which struggled to accurately differentiate between lesion regions and normal tissues, our method leveraged the curriculum learning strategy to enhance the feature representation capacity. This improvement enabled better perception of the target shape and texture, thereby resulting in the production of accurate segmentation masks and effective discrimination between the lesion regions and normal tissues.

Our method demonstrated significant advancements in semi-supervised medical image segmentation tasks, particularly in cases involving large-scale targets with low image clarity. These performance improvements have practical implications in the field of clinical medical-assisted diagnosis, wherein they can aide doctors in identifying lesion areas. By providing precise segmentation results, our method facilitates subsequent medical planning and treatment options, thereby leading to improved patient care. Nevertheless, it is crucial to acknowledge the limitations of our approach in certain scenarios. For instance, in high-resolution medical images or tasks involving small targets such as pulmonary nodules and micro-calcifications, our self-learning approach, which relies on inter-patch information, may not effectively enhance the model’s feature representation. These challenges need to be addressed to optimize the performance in such cases. Moreover, many medical video segmentation tasks that require semi-supervised methods pose additional challenges. Currently, our approach lacks effective utilization of the temporal context information between consecutive frames, which limits its applicability in these scenarios. Further research and development are required to incorporate temporal information and improve the performance in medical video segmentation. Another consideration is the training speed on large-volume datasets, which can be time-consuming and computationally expensive. Efforts should be directed toward optimizing the training efficiency without compromising the quality of the segmentation results. Furthermore, the extension of our patch grouping approach to 3D medical image tasks represents a significant challenge. Exploring innovative methodologies and techniques will be crucial to address this limitation and advance the field of 3D medical image segmentation.

While our method demonstrates remarkable performance improvements in semi-supervised medical image segmentation tasks, there are still challenges to overcome. By addressing the limitations mentioned above, we can further enhance the applicability and effectiveness of our approach in various medical imaging scenarios, thus ultimately benefiting both clinicians and patients.

## 5. Conclusions

In this study, our primary focus was to tackle the challenge of the limited availability of labeled medical image data in semi-supervised medical image segmentation. The scarcity of labeled data poses a significant hurdle, and, thus, we proposed a novel curriculum learning method that leverages patch-image-level contrastive learning. Our objective was to guide the segmentation model in learning pixel-level target features by progressively transitioning from easy to hard examples. This approach enhanced the model’s generalization ability and ultimately improved the quality of the segmentation results.

To achieve this, we introduced a curriculum consistency learning scheme that allowed the model to learn diverse feature representations, thereby enhancing its overall performance. Additionally, we incorporated a multi-scale contrastive constraint that facilitated model convergence during the training process. By effectively utilizing both the abundant un-labeled data and the limited labeled data, our framework dynamically explored the intrinsic relationships present across patches and images.

To validate the effectiveness of our proposed method, we conducted extensive experiments on two well-known datasets: the Kvasir-SEG polyp dataset and the ISIC 2018 skin lesion dataset. Through comprehensive metric evaluations and qualitative visualizations, we provided compelling evidence showcasing the superiority and efficacy of our approach compared to previous semi-supervised medical image segmentation methods. Furthermore, we performed meticulous ablation studies on both datasets to quantitatively measure the individual performance improvements contributed by each of the proposed modules within our method.

While there is still room for improvement and further development, particularly in handling large-scale medical data involving small targets and 3D medical image segmentation, our current method already offers invaluable assistance to medical professionals. It aids in target annotation and analysis tasks related to skin lesions, polyps, and similar scenarios, thereby facilitating more accurate diagnosis and the design of optimized medical treatment plans. The impact of our method on these practical applications is significant and has the potential to contribute to advancements in the field of medical imaging. 

## Figures and Tables

**Table 1:** Comparison of our approach with SOTA semi-supervised medical image segmentation methods on Kvasir-SEG dataset with 120 labeled images and 480 un-labeled images. Bold values represent the best performance.

Method	MAE	mIoU	Dice
U-net	8.16	57.08 ± 0.64	67.03 ± 0.87
UPRC	7.10	61.50 ± 0.71	70.83 ± 0.24
UAMT	7.40	59.43 ± 1.11	69.87 ± 0.61
CCT	6.77	64.33 ± 0.41	72.60 ± 0.44
CLCC	6.63	63.50 ± 0.16	73.63 ± 0.25
Ours (all)	**4.70**	**72.73** ± **0.08**	**81.47** ± **0.61**
Ours (w/o $L_{cc}$)	6.90	64.6 ± 0.66	73.46 ± 1.50
Ours (w/o $L_{msc}$)	5.33	70.33 ± 0.75	79.50 ± 0.90

**Table 2:** Comparison of our approach with SOTA methods for the ISIC 2018 dataset with 156 labeled images and 1400 un-labeled images. Bold values represent the best performance.

Method	MAE	mIoU	Dice
U-net	8.03	70.77 ± 1.16	79.2 ± 0.93
UPRC	7.23	71.83 ± 2.19	80.13 ± 2.21
UAMT	7.13	72.16 ± 2.10	80.70 ± 1.87
CCT	7.50	72.20 ± 0.16	81.40 ± 0.07
CLCC	6.90	74.00 ± 0.57	82.47 ± 0.73
Ours (all)	**6.53**	**74.50** ± **0.49**	**83.23** ± **0.59**
Ours (w/o $L_{cc}$)	7.37	71.20 ± 1.00	80.3 ± 1.56
Ours (w/o $L_{msc}$)	7.97	70.87 ± 0.74	79.6 ± 1.47

## Data Availability

The data presented in this study are available in [22,23].
